# Supplementary material for: Primary Healthcare Providers’ Views on Periodic COVID-19 Booster Vaccination for Themselves and Their Patients: A 2023 Nationwide Survey in Belgium
Source: Vaccines (Basel). 2024 Jul 3;12(7):740. doi: 10.3390/vaccines12070740 (PMC11281441; doi:10.3390/vaccines12070740)
Supplement: Supplementary file 1 [file vaccines-12-00740-s001.zip › Material_File_S1.pdf]

COVID-19 booster vaccination: side-effects & attitudes

The current survey consists of **two parts**.

Part 1 aims to collect data on your **side-effects** of COVID-19 vaccines in the past year.

Part 2 aims to assess your **attitudes** toward a periodic COVID-19 booster vaccine for yourself and your patients.

Thank you in advance for carefully completing this survey.

CHARMING
Coronavirus HuisARtsenpraktijk - MédecINe Générale

There are 48 questions in this survey.

Your side-effects of COVID-19 booster vaccination

This part will assess your side-effects of COVID-19 and influenza vaccination in the past year, and your symptoms in case of a COVID-19 infection.

1
Your personnal study code (C followed by 4 numbers; see our email sent by .... with your link to this questionnaire):

*

Please write your answer here:

2
What is your **current profession**?
*

Please choose **only one** of the following:

- ◯ General practitioner
- ◯ Training GP
- ◯ Nurse
- ◯ Physiotherapist
- ◯ Other [ ]

3 What is the **postal code** of your practice? *

❗ Only numbers may be entered in this field.
Please write your answer here:

[ ]

4
Have you been tested positive for COVID-19 since the last CHARMING testing period? (The last survey took place between 13/12/2021 and 05/01/2022)
*

Please choose **only one** of the following:

- ◯ Yes, before my last booster COVID-19 vaccine
- ◯ Yes, after my last booster COVID-19 vaccine
- ◯ No
- ◯ I do not remember
- ◯ I prefer not to answer

5
You stated that you were tested positive for COVID-19 since your last booster COVID-19 vaccinaton, **when was the positive sample taken ?**

*

Only answer this question if the following conditions are met:
Answer was 'Yes, before my last booster COVID-19 vaccine' at question '4 [Q00004]' (Have you been tested positive for COVID-19 since the last CHARMING testing period? (The last survey took place between 13/12/2021 and 05/01/2022))

Please enter a date:

6
You stated that you were tested positive for COVID-19 since your last booster COVID-19 vaccinaton, **did you have symptoms during this COVID-19 infection?**

*

Only answer this question if the following conditions are met:
Answer was 'Yes, before my last booster COVID-19 vaccine' at question '4 [Q00004]' (Have you been tested positive for COVID-19 since the last CHARMING testing period? (The last survey took place between 13/12/2021 and 05/01/2022))

❗ Choose one of the following answers
Please choose **only one** of the following:

- ◯ No
- ◯ Negligible
- ◯ Mild
- ◯ Moderate
- ◯ Severe
- ◯ I do not remember
- ◯ I prefer not to answer

7
You stated that you were tested positive for COVID-19 and have had symptoms, **for how many days did you experience the following symptoms?**

*

Only answer this question if the following conditions are met:
Answer was 'Yes, before my last booster COVID-19 vaccine' at question '4 [Q00004]' (Have you been tested positive for COVID-19 since the last CHARMING testing period? (The last survey took place between 13/12/2021 and 05/01/2022)) *and* Answer was 'Moderate' *or* 'Severe' at question '6 [Q00006]' (You stated that you were tested positive for COVID-19 since your last booster COVID-19 vaccinaton, did you have symptoms during this COVID-19 infection?)

Please choose the appropriate response for each item:

	**I didn't experience this symptom**	**1 day**	**2 days**	**3 days**	**4 days**	**5 days**	**6 days**	**7 days**	**8 days**	**9 days**	**10 days**	**>10 days**	**I do not rememb**
**Fever**	○	○	○	○	○	○	○	○	○	○	○	○	○
**Cough**	○	○	○	○	○	○	○	○	○	○	○	○	○
**Shortness of breath**	○	○	○	○	○	○	○	○	○	○	○	○	○
**Headache**	○	○	○	○	○	○	○	○	○	○	○	○	○
**Muscle pains**	○	○	○	○	○	○	○	○	○	○	○	○	○
**Fatigue**	○	○	○	○	○	○	○	○	○	○	○	○	○
**Other symptoms**	○	○	○	○	○	○	○	○	○	○	○	○	○

8 If you indicated that you had other symptoms in the previous question, what were those symptoms?

Only answer this question if the following conditions are met:
Answer was 'Yes, before my last booster COVID-19 vaccine' at question '4 [Q00004]' (Have you been tested positive for COVID-19 since the last CHARMING testing period? (The last survey took place between 13/12/2021 and 05/01/2022)) *and* Answer was 'Severe' *or* 'Moderate' at question '6 [Q00006]' (You stated that you were tested positive for COVID-19 since your last booster COVID-19 vaccinaton, did you have symptoms during this COVID-19 infection?)

Please write your answer here:

9

After the last CHARMING-survey, which of the following best describes how many COVID-19 vaccine doses you have received for yourself to date? **You can choose multiple doses** you received since the last CHARMING testing period.

*

Please choose **all** that apply:

- ☐ I have had the basic vaccination (two doses for Pfizer, AstraZeneca, Moderna … or one dose for Johnson & Johnson)
- ☐ I have had a third dose
- ☐ I have had a fourth dose
- ☐ I have had a fifth dose
- ☐ I have not received any COVID-19 vaccines
- ☐ I prefer not to answer

The last survey took place between 13/12/2021 and 05/01/2022.

10
When did you receive the THIRD dose of the vaccine ?

*

Only answer this question if the following conditions are met:
Answer was 'I have had a third dose' at question '9 [Q00009]' (After the last CHARMING-survey, which of the following best describes how many COVID-19 vaccine doses you have received for yourself to date? You can choose multiple doses you received since the last CHARMING testing period.)

Please enter a date:

11
Which vaccine did you receive ?

*

Only answer this question if the following conditions are met:
Answer was 'I have had a third dose' at question '9 [Q00009]' (After the last CHARMING-survey, which of the following best describes how many COVID-19 vaccine doses you have received for yourself to date? You can choose multiple doses you received since the last CHARMING testing period.)

Choose one of the following answers
Please choose **only one** of the following:

- ◯ Pfizer (Comirnaty)
- ◯ Moderna (Spikevax)
- ◯ I do not remember
- ◯ I prefer not to answer
- ◯ Other

12
Did you experience side-effects after receiving the THIRD dose ?
*

Only answer this question if the following conditions are met:
Answer was 'I have had a third dose' at question '9 [Q00009]' (After the last CHARMING-survey, which of the following best describes how many COVID-19 vaccine doses you have received for yourself to date? You can choose multiple doses you received since the last CHARMING testing period.   )

❗ Choose one of the following answers
Please choose **only one** of the following:

- ◯ No
- ◯ Negligible
- ◯ Mild
- ◯ Moderate
- ◯ Severe
- ◯ I do not remember
- ◯ I prefer not to answer

13
For how many days after receiving this THIRD dose of the vaccine did you experience the following side-effects ?

*

Only answer this question if the following conditions are met:
Answer was 'I have had a third dose' at question '9 [Q00009]' (After the last CHARMING-survey, which of the following best describes how many COVID-19 vaccine doses you have received for yourself to date? You can choose multiple doses you received since the last CHARMING testing period.) *and* Answer was 'Severe' *or* 'Moderate' at question '12 [Q00013]' (Did you experience side-effects after receiving the THIRD dose ?)

Please choose the appropriate response for each item:

	**I didn't experience this symptom**	**1 day**	**2 days**	**3 days**	**4 days**	**5 days**	**6 days**	**7 days**	**8 days**	**9 days**	**10 days**	**> 10 days**	**I don't rememb**
**Fever**	◯	◯	◯	◯	◯	◯	◯	◯	◯	◯	◯	◯	◯
**Cough**	◯	◯	◯	◯	◯	◯	◯	◯	◯	◯	◯	◯	◯
**Shortness of breath**	◯	◯	◯	◯	◯	◯	◯	◯	◯	◯	◯	◯	◯
**Headache**	◯	◯	◯	◯	◯	◯	◯	◯	◯	◯	◯	◯	◯
**Muscle pains**	◯	◯	◯	◯	◯	◯	◯	◯	◯	◯	◯	◯	◯
**Fatigue**	◯	◯	◯	◯	◯	◯	◯	◯	◯	◯	◯	◯	◯
**Other symptoms**	◯	◯	◯	◯	◯	◯	◯	◯	◯	◯	◯	◯	◯

14
If you indicated that you had other symptoms in the previous question, what were those symptoms?

Only answer this question if the following conditions are met:
Answer was 'I have had a third dose' at question '9 [Q00009]' (After the last CHARMING-survey, which of the following best describes how many COVID-19 vaccine doses you have received for yourself to date? You can choose multiple doses you received since the last CHARMING testing period.) *and* Answer was 'Moderate' *or* 'Severe' at question '12 [Q00013]' (Did you experience side-effects after receiving the THIRD dose ?)

Please write your answer here:

15
When did you receive the FOURTH dose of the vaccine ?

*

Only answer this question if the following conditions are met:
Answer was 'I have had a fourth dose' at question '9 [Q00009]' (After the last CHARMING-survey, which of the following best describes how many COVID-19 vaccine doses you have received for yourself to date? You can choose multiple doses you received since the last CHARMING testing period.)

Please enter a date:

16
Which vaccine did you receive?

*

Only answer this question if the following conditions are met:
Answer was 'I have had a fourth dose' at question '9 [Q00009]' (After the last CHARMING-survey, which of the following best describes how many COVID-19 vaccine doses you have received for yourself to date? You can choose multiple doses you received since the last CHARMING testing period.   )

❗ Choose one of the following answers
Please choose **only one** of the following:

- ◯ Pfizer (Comirnaty)
- ◯ Moderna (Spikevax)
- ◯ I do not remember
- ◯ I prefer not to answer
- ◯ Other ______

17
Did you experience side-effects after receiving the FOURTH dose ?
*

Only answer this question if the following conditions are met:
Answer was 'I have had a fourth dose' at question '9 [Q00009]' (After the last CHARMING-survey, which of the following best describes how many COVID-19 vaccine doses you have received for yourself to date? You can choose multiple doses you received since the last CHARMING testing period.   )

❗ Choose one of the following answers
Please choose **only one** of the following:

- ◯ No
- ◯ Negligible
- ◯ Mild
- ◯ Moderate
- ◯ Severe
- ◯ I do not remember
- ◯ I prefer not to answer

18
For how many days after receiving this FOURTH dose of the vaccine did you experience the following side-effects ?

*

Only answer this question if the following conditions are met:
Answer was 'I have had a fourth dose' at question '9 [Q00009]' (After the last CHARMING-survey, which of the following best describes how many COVID-19 vaccine doses you have received for yourself to date? You can choose multiple doses you received since the last CHARMING testing period.) *and* Answer was 'Severe' *or* 'Moderate' at question '17 [Q00018]' (Did you experience side-effects after receiving the FOURTH dose ?)

Please choose the appropriate response for each item:

	**I did not experience this symptom**	**1 day**	**2 days**	**3 days**	**4 days**	**5 days**	**6 days**	**7 days**	**8 days**	**9 days**	**10 days**	**>10 days**	**I do not rememb**
**Fever**	◯	◯	◯	◯	◯	◯	◯	◯	◯	◯	◯	◯	◯
**Cough**	◯	◯	◯	◯	◯	◯	◯	◯	◯	◯	◯	◯	◯
**Shortness of breath**	◯	◯	◯	◯	◯	◯	◯	◯	◯	◯	◯	◯	◯
**Headache**	◯	◯	◯	◯	◯	◯	◯	◯	◯	◯	◯	◯	◯
**Muscle pains**	◯	◯	◯	◯	◯	◯	◯	◯	◯	◯	◯	◯	◯
**Fatigue**	◯	◯	◯	◯	◯	◯	◯	◯	◯	◯	◯	◯	◯
**Other symptoms**	◯	◯	◯	◯	◯	◯	◯	◯	◯	◯	◯	◯	◯

19
If you indicated that you had other symptoms in the previous question, what were those symptoms?

Only answer this question if the following conditions are met:
Answer was 'I have had a fourth dose' at question '9 [Q00009]' (After the last CHARMING-survey, which of the following best describes how many COVID-19 vaccine doses you have received for yourself to date? You can choose multiple doses you received since the last CHARMING testing period.) *and* Answer was 'Severe' *or* 'Moderate' at question '17 [Q00018]' (Did you experience side-effects after receiving the FOURTH dose ?)

Please write your answer here:

20
When did you receive the FIFTH dose of the vaccine ?

*

Only answer this question if the following conditions are met:
Answer was 'I have had a fifth dose' at question '9 [Q00009]' (After the last CHARMING-survey, which of the following best describes how many COVID-19 vaccine doses you have received for yourself to date? You can choose multiple doses you received since the last CHARMING testing period.)

Please enter a date:

21
Which vaccine did you receive ?
*

Only answer this question if the following conditions are met:
Answer was 'I have had a fifth dose' at question '9 [Q00009]' (After the last CHARMING-survey, which of the following best describes how many COVID-19 vaccine doses you have received for yourself to date? You can choose multiple doses you received since the last CHARMING testing period.)

❗ Choose one of the following answers
Please choose **only one** of the following:

- ◯ Pfizer (Comirnaty)
- ◯ Moderna (Spikevax)
- ◯ I do not remember
- ◯ I prefer not to answer
- ◯ Other ____________

22

Did you experience side-effects after receiving the FIFTH dose ?

*

Only answer this question if the following conditions are met:
Answer was 'I have had a fifth dose' at question '9 [Q00009]' (After the last CHARMING-survey, which of the following best describes how many COVID-19 vaccine doses you have received for yourself to date? You can choose multiple doses you received since the last CHARMING testing period.)

Choose one of the following answers
Please choose **only one** of the following:

- No
- Negligible
- Mild
- Moderate
- Severe
- I do not remember
- I prefer not to answer

23
For how many days after receiving this FIFTH dose of the vaccine did you experience the following side-effects ?

*

Only answer this question if the following conditions are met:
Answer was 'I have had a fifth dose' at question '9 [Q00009]' (After the last CHARMING-survey, which of the following best describes how many COVID-19 vaccine doses you have received for yourself to date? You can choose multiple doses you received since the last CHARMING testing period.) *and* Answer was 'Severe' *or* 'Moderate' at question '22 [Q00023]' (Did you experience side-effects after receiving the FIFTH dose ?)

Please choose the appropriate response for each item:

	**I did not experience this symptom**	**1 day**	**2 days**	**3 days**	**4 days**	**5 days**	**6 days**	**7 days**	**8 days**	**9 days**	**10 days**	**>10 days**	**I do not rememb**
**Fever**	○	○	○	○	○	○	○	○	○	○	○	○	○
**Cough**	○	○	○	○	○	○	○	○	○	○	○	○	○
**Shortness of breath**	○	○	○	○	○	○	○	○	○	○	○	○	○
**Headache**	○	○	○	○	○	○	○	○	○	○	○	○	○
**Muscle pains**	○	○	○	○	○	○	○	○	○	○	○	○	○
**Fatigue**	○	○	○	○	○	○	○	○	○	○	○	○	○
**Other symptoms**	○	○	○	○	○	○	○	○	○	○	○	○	○

24
If you indicated that you had other symptoms in the previous question, what were those symptoms?

Only answer this question if the following conditions are met:
Answer was 'I have had a fifth dose' at question '9 [Q00009]' (After the last CHARMING-survey, which of the following best describes how many COVID-19 vaccine doses you have received for yourself to date? You can choose multiple doses you received since the last CHARMING testing period.) *and* Answer was 'Severe' *or* 'Moderate' at question '22 [Q00023]' (Did you experience side-effects after receiving the FIFTH dose ?)

Please write your answer here:

25
Are you vaccinated against flu for the 2022/2023 season?
*

Choose one of the following answers
Please choose **only one** of the following:

- ◯ Yes
- ◯ No
- ◯ I do not remember
- ◯ I prefer not to answer

26
Did you experience side-effects after receiving this influenza vaccine?

*

Only answer this question if the following conditions are met:
Answer was 'Yes' at question '25 [Q00026]' (Are you vaccinated against flu for the 2022/2023 season?)

❗ Choose one of the following answers
Please choose **only one** of the following:

- ◯ No
- ◯ Negligible
- ◯ Mild
- ◯ Moderate
- ◯ Severe
- ◯ I do not remember
- ◯ I prefer not to answer

27
For how many days after receiving this influenza vaccine did you experience the following side-effects?
*

Only answer this question if the following conditions are met:
Answer was 'Yes' at question '25 [Q00026]' (Are you vaccinated against flu for the 2022/2023 season?) *and* Answer was 'Severe' *or* 'Moderate' at question '26 [Q00027]' (Did you experience side-effects after receiving this influenza vaccine?)

Please choose the appropriate response for each item:

	I didn't experience this symptom	1 day	2 days	3 days	4 days	5 days	6 days	7 days	8 days	9 days	10 days	> 10 days	I do not rememb
**Fever**	○	○	○	○	○	○	○	○	○	○	○	○	○
**Cough**	○	○	○	○	○	○	○	○	○	○	○	○	○
**Shortness of breath**	○	○	○	○	○	○	○	○	○	○	○	○	○
**Headache**	○	○	○	○	○	○	○	○	○	○	○	○	○
**Muscle pains**	○	○	○	○	○	○	○	○	○	○	○	○	○
**Fatigue**	○	○	○	○	○	○	○	○	○	○	○	○	○
**Other symptoms**	○	○	○	○	○	○	○	○	○	○	○	○	○

28 If you indicated that you had other symptoms in the previous question, what were those symptoms?

Only answer this question if the following conditions are met:
Answer was 'Yes' at question '25 [Q00026]' (Are you vaccinated against flu for the 2022/2023 season?) *and* Answer was 'Severe' *or* 'Moderate' at question '26 [Q00027]' (Did you experience side-effects after receiving this influenza vaccine?)

Please write your answer here:

Your attitudes toward COVID-19 booster vaccination

This part will assess your attitudes toward annual COVID-19 vaccination for yourself and your patients.

29
In your role as a healthcare professional, are you ever involved in prescribing, administering, or recommending vaccines?

*

Please choose **only one** of the following:

- ◯ Yes
- ◯ No

30
How strongly do you agree or disagree with the following statements about the booster COVID-19 vaccines?
*

Please choose the appropriate response for each item:

	Strongly agree	Tend to agree	Tend to disagree	Strongly disagree	I don't know
**Overall, I think the COVID-19 booster vaccines are safe**	◯	◯	◯	◯	◯
**Overall, I think the COVID-19 booster vaccines are important**	◯	◯	◯	◯	◯
**Overall, I think the COVID-19 booster vaccines are effective at preventing me from getting infected with coronavirus/SARS-CoV-2/COVID-19**	◯	◯	◯	◯	◯
**Overall I think the COVID-19 booster vaccines are effective at preventing me from developing severe disease caused by COVID-19**	◯	◯	◯	◯	◯
**Overall, I think the COVID-19 booster vaccines are effective at preventing me from transmitting coronavirus/SARS-CoV-2/COVID-19 to others**	◯	◯	◯	◯	◯

	Strongly agree	Tend to agree	Tend to disagree	Strongly disagree	I don't know
**Overall, I think the COVID-19 booster vaccines are compatible with my religious, personal or philosophical beliefs**	◯	◯	◯	◯	◯

31

Would you accept **a periodic booster COVID-19 vaccine** if it was an official recommendation and you had already had all previous vaccines ?

*

Please choose **only one** of the following:

- ◯ Yes,definitely
- ◯ Unsure, but leaning towards yes
- ◯ Unsure, but leaning towards no
- ◯ No, definitely not

32
You mentioned that you were either unsure, but leaning towards yes or that you definitely want to receive a periodic COVID-19 booster vaccination. **Give the three main reasons from the list that best summarise your decision.**

Only answer this question if the following conditions are met:
Answer was 'Unsure, but leaning towards yes' *or* 'Yes,definitely' at question '31 [Q00032]' (Would you accept a periodic booster COVID-19 vaccine if it was an official recommendation and you had already had all previous vaccines ?)

Please number each box in order of preference from 1 to 16
Please choose at least 1 items.
Please choose no more than 3 items.

☐ To protect myself against getting infected with COVID-19/coronavirus/SARS-CoV-2

☐ To protect myself against getting seriously ill with COVID-19/coronavirus/SARS-CoV-2

☐ To protect my patients against getting seriously ill with COVID-19/coronavirus/SARS-CoV-2

☐ To protect people that I know personally (for example, a friend, a family member, colleague or patients) from getting infected

☐ To protect people that I know personally (for example, a friend, a family member, colleague or patients) from getting seriously ill with COVID-19/coronavirus/SARS-CoV-2

☐ To facilitate international travel

☐ To obtain domestic freedoms (going to restaurants, cafes, cinemas, sports events, night clubs, music events, domestic travel, etc)

☐ Because it was required or mandated (for example by your Gov-ernment, workplace, etc)

☐ To visit or use a healthcare service or facility (such as for medical help/treatment or to visit a care home)

☐ Because of pressure from my employer, friends, or family

☐ A healthcare professional recommended vaccinating

☐ Somebody that I know personally (for example, a friend, family member, or colleague) recommended vaccinating

☐ Somebody that I do not know personally recommended vaccinating (for example, a celebrity, public figure, etc)

☐ I don't want/wouldn't want to get sick so I can provide consistency in the practice for patients and colleagues

☐ None of the options

☐ Other

33 **If you mentioned the reason "other"** in the top three reasons why you would periodically be vaccinated with the COVID-19 booster vaccine, **please mention the reason here.**

Only answer this question if the following conditions are met:
Answer was 'Unsure, but leaning towards yes' *or* 'Yes,definitely' at question '31 [Q00032]' (Would you accept a periodic booster COVID-19 vaccine if it was an official recommendation and you had already had all previous vaccines ?)

Please write your answer here:

34
You mentioned that you were either unsure or that you definitely did not want to receive a periodic COVID-19 booster vaccination. **Give the three main reasons from the list that best summarise your decision.**

Only answer this question if the following conditions are met:
Answer was 'No, definitely not' *or* 'Unsure, but leaning towards no' at question '31 [Q00032]' (Would you accept a periodic booster COVID-19 vaccine if it was an official recommendation and you had already had all previous vaccines ?)

Please number each box in order of preference from 1 to 20
Please choose at least 1 items.
Please choose no more than 3 items.

☐ Somebody that I know personally had an adverse reaction to a COVID-19 vaccine (for example, a friend, family member, or colleague)

☐ Somebody that I do not know had an adverse reaction to a COVID-19 vaccine (for example, a celebrity, public figure, etc)

☐ I am worried about COVID-19 vaccine side effects

☐ I want to choose which COVID-19 vaccine I have

☐ A medical professional has recommended against receiving a COVID-19 vaccine

☐ Friends or family have recommended against receiving a COVID-19 vaccine

☐ I was never advised or invited to be vaccinated

☐ I do not feel that COVID-19 presents a serious risk to me

☐ I do not think that COVID-19 vaccines are effective at pre-venting getting infected with COVID-19/coronavirus/SARS-CoV-2

☐ I do not think that COVID-19 vaccines are effective at pre-venting me from getting seriously ill with COVID-19

☐ I have concerns over vaccine passports or vaccine mandates

- [ ] I have been previously infected with coronavirus/SARS-COV-2 and have natural immunity
- [ ] I do not think COVID-19 vaccines have been sufficiently tested
- [ ] I want to get vaccinated, but I find/found it hard to receive a COVID-19 vaccine (for example, I could not make or attend an appointment)
- [ ] I already received at least one dose of COVID-19 vaccine and I had an adverse reaction
- [ ] I have concerns about the ingredients of COVID-19 vaccines
- [ ] I am against all types of vaccination
- [ ] I have a medical exemption
- [ ] None of the options
- [ ] Other

35

If you mentioned the reason "other" in the top three reasons why you would not periodically be vaccinated with the COVID-19 booster vaccine, **please mention the reason here**

Only answer this question if the following conditions are met:
Answer was 'No, definitely not' *or* 'Unsure, but leaning towards no' at question '31 [Q00032]' (Would you accept a periodic booster COVID-19 vaccine if it was an official recommendation and you had already had all previous vaccines ?)

Please write your answer here:

36
To what extent do you agree that a COVID-19 booster vaccination should be administered periodically to...:

*

Please choose the appropriate response for each item:

	Strongly agree	Tend to agree	Tend to disagree	Strongly disagree	I don't know
**All primary health care providers**	◯	◯	◯	◯	◯
**Certain groups of primary health care providers who have co-morbidities defined as risk factors for severe COVID-19 disease**	◯	◯	◯	◯	◯
**The whole population**	◯	◯	◯	◯	◯
**Those patients who have co-morbidities defined as risk factors for severe COVID-19 disease**	◯	◯	◯	◯	◯

37

How likely are you to periodically recommend a COVID-19 booster vaccine to eligible* patients?

*

Please choose the appropriate response for each item:

Highly likely	Somewhat likely	Somewhat unlikely	Highly unlikely	I don't know
○	○	○	○	○

**for information, by the 01/12/2022 the Ministers of Health have decided, based on the advice of the Superior Health Council and the Vaccination Task Force, to give everyone aged 18 and older the opportunity to get an ‘autumn booster’. ‘, this time with a adapted vaccine. The Fall Booster is highly recommended for those working in the healthcare sector and for those most at risk for severe COVID-19: everyone from 50 years old and people with weakened immunity*

38

You stated that you would **be unlikely to periodically recommend a COVID-19 booster vaccine** to patients. Which of the following best describes the **reason(s) why?** (Please select all that apply)

*

Only answer this question if the following conditions are met:
Answer was 'Highly unlikely' *or* 'Somewhat unlikely' at question '37 [Q00035]' (How likely are you to periodically recommend a COVID-19 booster vaccine to eligible* patients? ())

Please choose **all** that apply:

- [ ] I do not think my patients are at risk of contracting the virus
- [ ] I am confident that other effective treatments will be available soon
- [ ] I do not yet know enough about the COVID-19 booster vaccine to recommend it
- [ ] The approval/development of the vaccine and COVID-19 booster vaccine may be rushed, and the vaccine may not have been sufficiently tested
- [ ] I think that vaccines can make you get the disease they are supposed to protect you from
- [ ] I don't know enough about its potential safety yet
- [ ] I don't know enough about its potential effectiveness yet
- [ ] I don't think my patients would be seriously ill if they got the virus
- [ ] I prefer to wait for other people to get the vaccine first
- [ ] The vaccine could have serious side effects
- [ ] The risks associated with the vaccine outweigh the benefits
- [ ] COVID-19 is not very common
- [ ] COVID-19 is not dangerous
- [ ] Adjuvants in the vaccine could be dangerous
- [ ] Patients do not want to receive the COVID-19 vaccine
- [ ] Colleagues have told me that they do not recommend the vaccine
- [ ] I prefer to recommend natural alternatives
- [ ] I don't know
- [ ] Other: ____________

39
To what extend do you agree with COVID-19 booster vaccination administration by pharmacists ?
*

Please choose the appropriate response for each item:

Strongly agree	Tend to agree	Tend to disagree	Strongly disagree	I don’t know
○	○	○	○	○

40
If you would suggest something else regarding who should administer the COVID-19 booster vaccine, please state it here:

Please write your answer here:

41
Do you think that administration of COVID-19 booster vaccinations by pharmacists will reduce your workload?

*

Please choose **only one** of the following:

- ◯ Yes, I think it will reduce my workload
- ◯ No, I don't think it will reduce my workload
- ◯ I don’t know

42
Do you believe that it is **your role to encourage your patients to get vaccinated** even if they are hesitants?

*

Please choose the appropriate response for each item:

**Strongly agree**	**Tend to agree**	**Tend to disagree**	**Strongly disagree**	**I don't know**
◯	◯	◯	◯	◯

43
In general, do you feel comfortable giving explanations to your patients about ... ?
*

Please choose the appropriate response for each item:

	Very comfortable	Somewhat comfortable	Somewhat uncomfortable	Not at all comfortable	I don't know
**1. The value of vaccines**	◯	◯	◯	◯	◯
**2. The safety of vaccines**	◯	◯	◯	◯	◯
**3. The role of adjuvants**	◯	◯	◯	◯	◯

44
How strongly do you agree or disagree with each of the following statements about **COVID-19 vaccines ?**
*

Please choose the appropriate response for each item:

	Strongly agree	Tend to agree	Tend to disagree	Strongly disagree	I don't know
**The government provides honest information regarding COVID-19 vaccination**	○	○	○	○	○
**The government is committed to protecting the public from COVID-19**	○	○	○	○	○
**The government's actions in response to COVID-19 are in my personal best interest.**	○	○	○	○	○
**I am satisfied with the way the government has handled COVID-19 vaccination in my country**	○	○	○	○	○
**Health authorities provide reliable information about COVID-19**	○	○	○	○	○
**International organisations (e.g. World Health Organisations, European union) provide reliable information about COVID-19**	○	○	○	○	○

	Strongly agree	Tend to agree	Tend to disagree	Strongly disagree	I don't know
Health professionals (such as doctors, nurses, pharmacists, etc) provide reliable information about COVID-19	◯	◯	◯	◯	◯
I can find reliable information about COVID-19 in the media (television, radio, newspapers)	◯	◯	◯	◯	◯
I can find reliable information about COVID-19 on the internet or social media	◯	◯	◯	◯	◯
Compared to before the COVID-19 pandemic, I feel more informed about vaccination in general	◯	◯	◯	◯	◯
Compared to before the COVID-19 pandemic, I am more confident generally in the safety of vaccines	◯	◯	◯	◯	◯
Compared to before the COVID-19 pandemic, I am more confident generally in the importance of vaccines	◯	◯	◯	◯	◯
Compared to before the COVID-19 pandemic, I am more confident generally in the effectiveness of vaccines	◯	◯	◯	◯	◯

	Strongly agree	Tend to agree	Tend to disagree	Strongly disagree	I don't know
**I can get reliable information about COVID-19 from a complimentary or alternative medical practitioner (e.g. Osteopath, homeopath, naturopath)**	◯	◯	◯	◯	◯

45
How much do you agree to have a seasonal Flu vaccination administered for ... :

*

Please choose the appropriate response for each item:

	Strongly agree	Tend to agree	Tend to disagree	Strongly disagree	I don’t know
**All primary health care provider**	◯	◯	◯	◯	◯
**Certain group of primary health care provider who belong to risk groups with regards to severe manifestations of flu infection**	◯	◯	◯	◯	◯
**The whole population**	◯	◯	◯	◯	◯
**Those patients who belong to risk groups with regards to severe manifestations of flu infection**	◯	◯	◯	◯	◯

46
Please answer the following relating to how you feel about COVID-19 vaccines.
*

Please choose the appropriate response for each item:

	Strongly disagree	Disagree	Neither agree or disagree	Agree	Strongly agree
**I feel/felt a sense of choice and freedom in whether to have a vaccine**	○	○	○	○	○
**I feel/felt that my decision whether to vaccinate reflects what I really want**	○	○	○	○	○
**I feel/felt forced or coerced to get vaccinated**	○	○	○	○	○
**I feel/felt that I will be 'punished' if I didn't get vaccinated**	○	○	○	○	○
**I feel/felt confident that I could get vaccinated if I wanted to**	○	○	○	○	○
**I feel/felt capable of getting vaccinated if I wanted to**	○	○	○	○	○
**I have/had serious doubts about whether I could get vaccinated if I wanted**	○	○	○	○	○
**I feel/felt that it would be difficult for me to get vaccinated if I wanted to**	○	○	○	○	○

	Strongly disagree	Disagree	Neither agree or disagree	Agree	Strongly agree
**I feel/felt that the official authorities care about me**	◯	◯	◯	◯	◯
**I feel/felt that the official authorities understand / understood my needs**	◯	◯	◯	◯	◯
**I feel/felt excluded by official authorities**	◯	◯	◯	◯	◯
**I feel/felt that the official authorities are/were cold and distant**	◯	◯	◯	◯	◯

47 Which of the following statement would you agree with the most? *

Only answer this question if the following conditions are met:
Answer was 'I have had a fifth dose' *or* 'I have had a fourth dose' *or* 'I have had a third dose' at question '9 [Q00009]' (After the last CHARMING-survey, which of the following best describes how many COVID-19 vaccine doses you have received for yourself to date? You can choose multiple doses you received since the last CHARMING testing period.)

Please choose **only one** of the following:

◯ I am relieved since I have had the last COVID-19 booster vaccine

◯ I regret having received the last COVID-19 booster vaccine

◯ I am angry to have received the last COVID-19 booster vaccine

◯ None of the above

48
Would you be willing to be contacted again for an in-depth follow-up interview ?
*

Please choose **only one** of the following:

- ◯ Yes
- ◯ No

Thank you very much for completing this questionnaire!

The CHARMING study team

CHARMING
Coronavirus HuisARtsenpraktijk - MédecINe Générale

Submit your survey.
Thank you for completing this survey.
