# Supplementary material for: Primary Healthcare Providers’ Views on Periodic COVID-19 Booster Vaccination for Themselves and Their Patients: A 2023 Nationwide Survey in Belgium
Source: Vaccines (Basel). 2024 Jul 3;12(7):740. doi: 10.3390/vaccines12070740 (PMC11281441; doi:10.3390/vaccines12070740)
Supplement: Supplementary file 1 [file vaccines-12-00740-s001.zip › Material_File_S2.pdf]

GENERAL THEME	THEME WITH PRECISIONS
GP	
	GP/NURSE
	GP/OCCUPATIONAL MEDICINE
	GP/PREVENTION CENTER
	GP/PREVENTION
	GP/SUPERVISION
	GP/NURSE SUPERVISED BY GP
	GP/TECHNICAL
	GP/WITH FLU VACCINE
NURSE	
	NURSE/GENERAL PRACTICE
	NURSE/GP
	NURSE/SUPERVISED BY GP
PHARMACIST	
	PHARMACIST WITH CONDITION
	PHARMACIST/COLLABORATION WITH GP
	PHARMACIST/GP
	PHARMACIST/PREVENTIVE CENTER
	PHARMACIST/SUPERVISED BY GP
	PHARMACIST/TECHNICAL
	PHARMACIST/WITH FLU VACCINE
CENTER	
	CENTER/GENERAL PRACTICE
	CENTER/GP
	CENTER/WITH FLU VACCINE
	CENTER/WITH FLU VACCINE/TECHNICAL
	CENTER/NOT GP PRACTICE
	GP/CENTER/NURSE
	NURSE/CENTER/WITH FLU
	OCCUPATIONAL MEDICINE/CENTER/NURSING HOME
	PREVENTIVE CENTER
COLLABORATION	
GENERAL PRACTICE	
	GENERAL PRACTICE /WITH FLU VACCINE
	GENERAL PRACTICE/GP
	GENERAL PRACTICE/TECHNICAL
	GENERAL PRACTICE/WITH FLU VACCINE/TECHICAL DOSE
	GENERAL PRACTICE/WITH NURSE
OCCUPATIONAL MEDICINE	
NURSING HOME	
NOT GP	
	NOT GP/COLLABORATION
	NOT GP/OCCUPATIONAL MEDICINE
	NOT GP/ORGANISATION
	NOT GP/TECHNICAL

NOT SHOULD BUT POSSIBILITY	
NOT PHARMACISTS	
TECHNICAL	
	TECHNICAL DOSE
	TECHNICAL DOSE/WITH FLU VACCINE
	UNIVERSAL TOOL TO RECORD ALL VACCINATIONS
	TRAINED VOLUNTEER
	WITH FLU VACCINE
