# Supplementary material for: Primary Healthcare Providers’ Views on Periodic COVID-19 Booster Vaccination for Themselves and Their Patients: A 2023 Nationwide Survey in Belgium
Source: Vaccines (Basel). 2024 Jul 3;12(7):740. doi: 10.3390/vaccines12070740 (PMC11281441; doi:10.3390/vaccines12070740)
Supplement: Supplementary file 1 [file vaccines-12-00740-s001.zip › Table_S1.pdf]

Supplementary Table S1: Baseline characteristics based on type of job for 1644 Belgian primary healthcare providers (PHCPs) responding to the survey about their views on periodic COVID-19 booster vaccine for themselves and their patients (between 17/02/2023 to 07/03/2023).

			Gender		Region			Practice size			
		age (median)	men (N)	woman (N)	Brussels (N)	Flanders (N)	Wallonia (N)	Solo (N)	Duo (N)	Group (N)	Big (N)
Type of job	GP	47	501	889	104	1014	272	312	227	338	499
	Nurse	41	5	61	2	47	17	4	2	6	54
	Other	50,5	22	84	13	73	20	15	14	12	57
	physiotherap	37	8	27	4	20	11	5	4	1	23
	Training GPs	27	20	27	5	36	6	3	11	10	23
