# Supplementary material for: Primary Healthcare Providers’ Views on Periodic COVID-19 Booster Vaccination for Themselves and Their Patients: A 2023 Nationwide Survey in Belgium
Source: Vaccines (Basel). 2024 Jul 3;12(7):740. doi: 10.3390/vaccines12070740 (PMC11281441; doi:10.3390/vaccines12070740)
Supplement: Supplementary file 1 [file vaccines-12-00740-s001.zip › Table_S2.pdf]

Supplementary Table S2: Odds of unwillingness to get a periodic COVID-19 booster vaccine by age categories (N = 1644).

	Willingness to get a periodic booster	Unwillingness to get a periodic booster	Unadjusted OR [95% CI]	Adjusted OR [95% CI]
	N = 1414	N = 230		
**Age categories (years)**				
≤ 30	156	16	1	1
31-40	393	83	**2.06 [1.20, 3.75]**	**2.52 [1.34, 5.09]**
41-50	266	54	**1.98 [1.12, 3.68]**	**2.46 [1.27, 5.08]**
51-60	251	36	1.40 [0.76, 2.67]	1.61 [0.80, 3.44]
>60	326	36	1.08 [0.59, 2.05]	1.46 [0.71, 3.14]
NA	22	5	NA	NA

Willingness vs unwillingness to get a periodic COVID-19 booster vaccine was determined as followed; PHCP who reported "yes, definitely" or "unsure, but leaning towards yes" to the question "Would you accept a periodic booster COVID-19 vaccine if it was an official recommendation and you had already had all previous vaccines?" were determined as willing to get a periodic booster. PHCP who answered "unsure, but leaning towards no" and "no, definitely" were considered as unwilling to get it. Profiles are distributed by age categories ≤30, 31-40, 41-50, 51-60 and >60. Data are shown as unadjusted odds ratio (OR) with 95% confidence interval (95%CI) and OR, adjusted for Gender, Region, Type of job and Practice size (multivariate analysis), with 95%CI. The ORs are estimated based on a logistic regression analysis.
