# Supplementary material for: Primary Healthcare Providers’ Views on Periodic COVID-19 Booster Vaccination for Themselves and Their Patients: A 2023 Nationwide Survey in Belgium
Source: Vaccines (Basel). 2024 Jul 3;12(7):740. doi: 10.3390/vaccines12070740 (PMC11281441; doi:10.3390/vaccines12070740)
Supplement: Supplementary file 1 [file vaccines-12-00740-s001.zip › Table_S3.pdf]

Supplementary Table S3: Odds of unwillingness to recommend a periodic COVID-19 booster vaccine for their patients as a function of individual characteristics (N = 1643).

	Willingness to recommend a periodic booster	Unwillingness to recommend a periodic booster	Unadjusted OR [95% CI]	Adjusted OR [95% CI]
	N = 1540	N = 103		
				**Model 1**
**Age (years)**			1.01 [0.99, 1.02]	1 [0.99, 1.02]
Median (IQR)	46	45		
**Gender**				
Female (ref)	1016	72	1	1
Male	524	31	0.94 [0.58, 1.5]	1.07 [0.65, 1.72]
**Region**				
Brussels	112	16	**3.15 [1.58, 5.89]**	**2.81 [1.43, 5.26]**
Flanders (ref)	1135	54	1	1
Wallonia	293	33	**2.35 [1.39, 3.89]**	**2.55 [1.55, 4.17]**
**Type of job**				
General practitionner	1329	60	**1**	1
Training GP	46	1	0.58 [0.03, 2.75]	0.50 [0.03, 2.48]
Nurse	61	5	2.39 [0.81, 5.72]	1.92 [0.63, 4.78]
Physiotherapist	25	10	**7.65 [2.71, 18.8]**	**8.82 [3.70, 19.9]**
Other	79	27	**7.5 [4.2, 13]**	**7.75 [4.37, 13.5]**
**Practice size**				
Solo (ref)	318	20	1	1
Duo	240	18	1.24 [0.59, 2.56]	1.33 [0.64, 2.73]
Group	351	16	0.85 [0.41, 1.75]	1.09 [0.51, 2.32]

Big	608	48	1.12<br>[0.62, 2.12]	1.12<br>[0.62, 2.10]
NA	23	1		
				**Model 2**
**Side effects related to influenza vaccination**				
No effects (ref)	945	30	1	1
Negligible	295	9	0.95<br>[0.4, 2.02]	0.85<br>[0.34, 2.00]
Mild	50	4	**1.46**<br>**[0.23, 5.07]**	0.93<br>[0.20, 3.45]
Moderate	5	2	17.1<br>[2.3, 91.8]	4.85<br>[0.40, 40.5]
Severe	1	0	NA	NA
I do not remember	2	0	NA	NA
**Number of booster received since last CHARMING testing**				
Zero	64	33	1	1
One	224	29	**0.26**<br>**[0.13, 0.51]**	0.46<br>[0.11, 1.86]
Two	1135	40	**0.1**<br>**[0.05, 0.17]**	1.45<br>[0.44, 5.17]
Three	112	1	**0.02**<br>**[0, 0.12]**	0.89<br>[0.04, 7.24]
Other	5	0	NA	NA
**Confidence in vaccination**				
Yes (ref)	1403	33	1	1
No	137	70	**25.5**<br>**[15.6, 42.3]**	**4.51**<br>**[1.80, 11.2]**
**Implication in vaccination as a healthcare worker**				
Yes (ref)	1482	68	1	1
No	58	35	**10.9**<br>**[6.25, 18.8]**	1.74<br>[0.51, 5.2]
**Willingness to get a booster for themselves**				
Yes	1383	30	1	1

No	157	73	**22 [13.5, 36.8]**	**5.89 [2.28, 15.1]**
**Healthcare professional's views regarding their role to encourage patients to get vaccinated**				
Strongly agree (ref)	759	9	1	1
Tend to agree	680	29	**3.19 [1.53, 7.27]**	**4.23 [1.33, 18.7]**
Tend to disagree	69	23	**25.8 [11.4, 62.6]**	**22.3 [5.95, 108]**
Strongly disagree	13	25	**166 [63.5, 480]**	**33.3 [8.70, 231]**
I don't know	19	17	**49.9 [17.3, 146]**	**62.6 [13.3, 355]**

Willingness vs unwillingness to recommend a periodic COVID-19 booster vaccine was determined as followed; PHCP who reported "Highly likely" or "somewhat likely" to the question "How likely are you to periodically recommend a COVID-19 vaccine to eligible patients ?" were determined as willing to recommend a periodic booster. PHCP who answered "I don't know", "somewhat unlikely" and "Highly unlikely" were considered as unwilling to recommend it. Profiles are distributed by individual characteristics in the first model (**model 1**): age (median), gender, region, type of job with jobs divided into general practitioner (GP), training GP, nurse, physiotherapist and other job and practice size (with solo being one employee, duo being two employee, group practice being maximum seven employee and big being more than seven employee in the practice). In the second model (**model 2**), participants' experiences are taking into account; self-reported side effects related to last influenza vaccination, number of booster received since the last charming testing that took place 13/12/2021 and 05/01/2022, confidence in vaccination based on confidence in vaccination based on positive responses for the three statements concerning the safety, importance and efficacy of booster shots against the development of severe forms of COVID-19, implication in vaccination as a healthcare worker, PHCP's views of their role in encouraging eligible patients to receive a booster when asking the question "Do you believe that it is your role to encourage your patients to get vaccinated even if they are hesitant ?" and their willingness to get a COVID-19 vaccine booster for themselves with PHCP who reported "yes, definitely" or "unsure, but leaning towards yes" to the question "Would you accept a periodic booster COVID-19 vaccine if it was an official recommendation and you had already had all previous vaccines ?" classified as willing to get a periodic booster (Yes). PHCP who answered "unsure, but leaning towards no" and "no, definitely" were considered as unwilling to get it (No). Data are shown as unadjusted odds ratio (OR) with 95% confidence interval (95%CI) and adjusted OR for all covariates (multivariate analysis) with 95%CI. The ORs are estimated based on logistic regression analysis.
