# Supplementary figures and images for: Primary Healthcare Providers’ Views on Periodic COVID-19 Booster Vaccination for Themselves and Their Patients: A 2023 Nationwide Survey in Belgium
Source: Vaccines (Basel). 2024 Jul 3;12(7):740. doi: 10.3390/vaccines12070740 (PMC11281441; doi:10.3390/vaccines12070740)

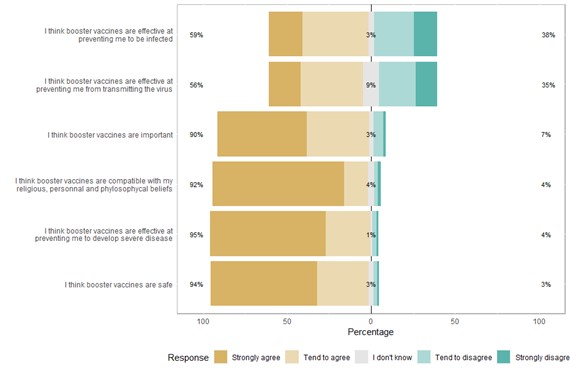

Supplement: Supplementary file 1 [file vaccines-12-00740-s001.zip › Figure_S1.jpg]

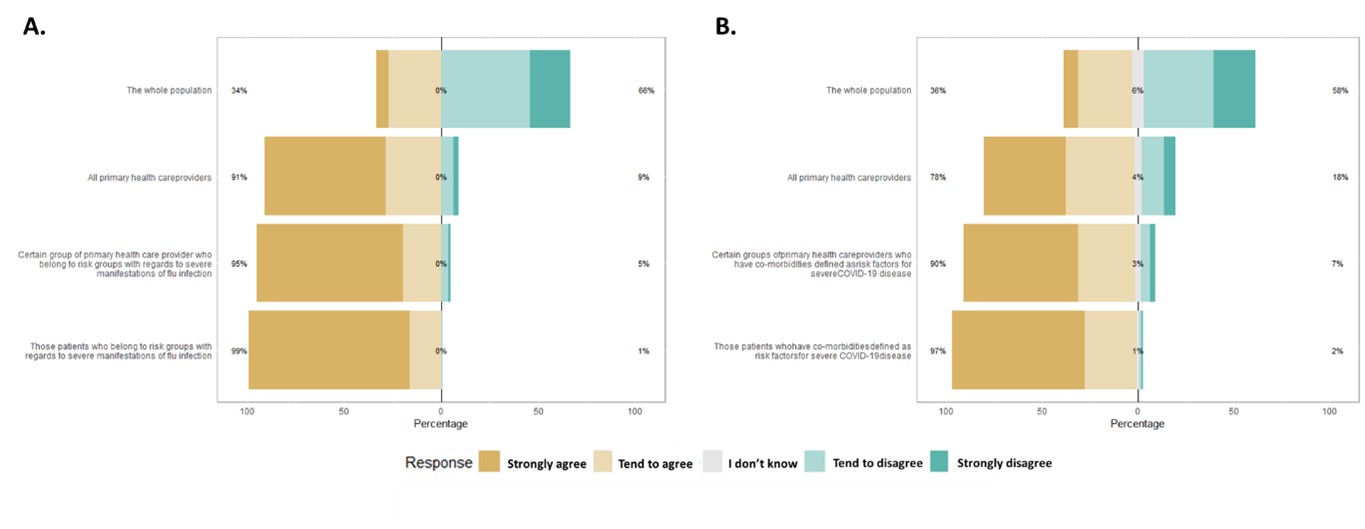

Supplement: Supplementary file 1 [file vaccines-12-00740-s001.zip › Figure_S2.jpg]

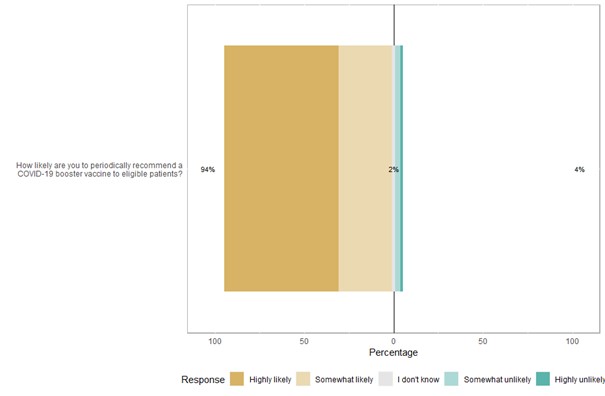

Supplement: Supplementary file 1 [file vaccines-12-00740-s001.zip › Figure_S3.jpg]
